# Editorial: Neuromanagement and Neuromarketing

**DOI:** 10.3389/fpsyg.2022.864566

**Published:** 2022-03-08

**Authors:** Vincenzo Russo, Qingguo Ma, Jesper Clement, Jia Jin, Tao Liu, Margherita Zito

**Affiliations:** ^1^Department of Business, Law, Economics and Consumer Behaviour “Carlo A. Ricciardi”, Università IULM, Milan, Italy; ^2^School of Management, Zhejiang University, Hangzhou, China; ^3^Department of Marketing, Copenhagen Business School, Frederiksberg, Denmark; ^4^School of Business and Management, Shanghai International Studies University, Shanghai, China

**Keywords:** neuromanagement, neuromarketing, neuromarketing tools, neuro-physiological, consumers' decisions, organizational psychology

This editorial summarizes the contributions to the Frontiers Research Topic on “Neuromanagement and Neuromarketing” with the aim to disseminate knowledge to advance research in these fields, and to explore the connections with the main theories and research approaches in the field of Organizational Psychology.

According to recent studies on consumers' behaviors and decision processes, measurements based on the registration of neuro-physiological parameters result in objective data, neuroscience applied to marketing can reveal what is happening in the brain in response to stimuli from advertising increasing our understanding of the neural mechanisms involved in buying decisions and emotional processes. The neuroscientific and marketing techniques are recognized to be able to analyze the real and unconscious effect of marketing stimuli. Liu et al. demonstrated the positive impact of reviewers' disclosure of personal review record on consumers' purchase decisions. The study is very interesting in the current era of electronic commerce. The reviewers' self-disclosure seems to have a positive impact on consumers' perception of source credibility, which in turn shapes consumer willingness to accept certain messages as well as their willingness to buy. The authors used the event-related potentials (ERPs) technique into marketing related research about the effect of reviewers' self-disclosure of personal review record on consumers' purchase decision making, analyzing the impact of source credibility of OCRs (online consumer reviews) on consumers' decisions. This research represents one of the first studies highlighting the role of personal review record in consumer behavior. Finally, the authors used neuromarketing tools, combining behavioral and ERPs approaches, to understand how self-disclosure of personal review record influences consumer information processing and decision-making.

Michela Balconi et al. evaluated the presence of distinct cortical brain oscillations in consumers' brain while navigating in a store and the effects of the specific role of touch on the customer experience. The lack of studies investigating the psychological dimensions and emotional aspects involved in sensory consumers' experience in-store, considering the effect of the touch, by employing a neuroscientific approach makes this work of great interest. This study suggested the possible usefulness of the Beta band on the right frontal hemisphere, analyzing with an EEG to measure affective states and higher-order cognitive sensory aspects more directly in a wide range of areas of interest where touch is involved. The presence of beta band suggests a cognitive state of sustained attention and enhanced network activity of higher-order somatosensory areas encoding perhaps the sensory aspects of the stimuli.

Duan et al. used a Functional near-infrared spectroscopy (fNIRS) to explore the neural correlates of consumers' purchase decision on different cross-culture marketing strategies. This methodology is a very promising brain imaging modality for neuromarketing research. The authors used this non-invasive technology by monitoring the regional cerebral hemoglobin concentration changes to analyze the different brain activation in males and females in relation to the cross-culture marketing strategies of the transnational brands. They simulated a virtual purchase scenario and measured the behavioral and neural responses toward two types of advertisements of different cross-culture marketing strategy. Findings suggest cognitive and emotional differences between men and women in purchase decision making toward different cross-culture marketing strategy: women showed higher purchase rate when watching the original culture advertisements than the mixed culture advertisements; man did not show significant preference between these two types. Neuromarketing approaches allow researchers to better understand complex purchase decision phenomena and make more comprehensive the assessment of marketing strategies, by analyzing the underlying neurobiology which are neglected or unavailable in traditional behavioral studies.

To understand responses to stimuli that are seen often, such as daily commodities, we need research tools able to detect the non-conscious reactions that are impossible for people to put words on. Utilizing the neuromarketing toolbox with a variety of neuro- and bio-metric techniques has shown how this approach to research brings new insights to people's reaction to daily stimuli. Yuan et al. utilized EEG to outline the impact from the color on warning signs that we see daily, to clarify the non-conscious effect of the color. Previous research on this issue based on verbal or behavioral responses but were not able to explain how the people and especially their brain responded spontaneously to warning signs in different colors. The non-conscious responses to different designs that we see in our daily life is central to research within consumer neuroscience. Alvino et al. proved benefits in an EEG based research design, to find the correlation between different designs and individual preferences. Predicting how designs of wine labels impacted early preferences of what the consumers were exposed to, would not have been possible by any traditional research tools in our marketing research toolbox. Recent research with neuromarketing show how specific design or changes in designs can change decisions. Russo et al. (A) and Russo et al. (B) emphasize the value of knowing more about how and what consumers look at local fish product and how the label design can influence purchase. Findings from this research would also be relevant for other food industry categories knowing how to design labels and others information given on packaging. From other research (Clement et al., 2017) we know that certain product characteristics and intrinsic values that are essential for band perception and brand equity do not get the attention needed.

As for the issue of tools and methodology, neuromarketing techniques show the potential to promote our understanding of consumer behavior. Mandolfo and Lamberti compared four research methods investigating impulsive buying, including quantitative self-reports, laboratory investigations, fieldwork observations, and qualitative interviews. They demonstrated that self-reports and interviews are effective to assess the cognitive facet of impulse purchasing, while laboratory investigations and fieldwork observations are appropriate to examine the conative and visceral facets of impulsive buying. Even though, however, the authors further identified two limitations in traditional approaches, concerning over-reliance on self-reports and lack of real-time assessment of cognitive and affective processes during impulsive buying, asking for complementary methods such as psychophysiological and neuroimaging tools. In this vein, Mauri et al. verified effectiveness of the psychophysiological techniques of implicit association test and emotional facial expressions for the assessment of user experience while navigating website, emphasizing importance of the emotional impact raised by website. Focusing on neuroscience approaches, Pei and Li reviewed literature using EEG-based affective computing technique in marketing and pointed out a promising avenue for investigation of affective states of consumers. In addition, the authors also called for attention to interactions among multiple customers. In line with this idea, Leeuwis et al. examined moment-to-moment neural similarity across subjects using EEG when they listen to music and validated that neural synchrony carries high predictive value for popularity of music. Yu et al. further demonstrated that in live streaming shopping context a broadcaster with strong passion and preparedness could enhance neural synchrony across consumers. Taken together, these articles have shown that neuromarketing techniques are valuable complements to traditional research methods in marketing by providing additional, often less subjective, and in-the-moment information about consumption decisions and interactive experiences.

Finally, neuroscience tools were applied also in organizational studies. The issue of neuromanagement considered new perspectives for the work context and dynamics.

Balconi and Fronda offer an important support describing the hyperscanning paradigm, consisting in the simultaneous recording of the cerebral activity of two or more subjects involved in social and interpersonal tasks, useful to deepen human interactions, and employed to understand exchanges in the managerial context and interactions between leaders and employees. This allowed to investigate the neural mechanisms of synthonization associated to leadership style, exploring interpersonal brain mechanisms generated by social interactions.

Leadership and interactions with followers, has been detected considering consciousness by Psychogios and Dimitriadis, in the framework of the social brain theory, underlying the mutual dependent relations, and adding the concept of *Homo Relationalis*, suggesting that leaders are social brain constructed phenomenon, requiring an understanding of the human brain as a social organ. Authors indicates different cognitive styles to understand the balance of leader/follower in a person, influencing relations and decisions.

The study by Johannesen and Zak, considered trust and company's purpose which enhance job tenure, job and life satisfaction, productivity, and decrease stress. To understand trust in organization, the authors considered studies suggesting that the neurochemical oxytocin is released in the brain after positive interactions. Through the measurement of employees' neurophysiology, motivation, and productivity, they identified eight behaviors though which organizations can affect trust. To quantify organizational trust the eight factors were operationalized in an OXYTOCIN measure, showing how trust and purpose can be key factors for productivity, satisfaction, health and reduced turnover, competitive points for organizations.

Finally, the study by Zito et al. focused on job assessment and neuroscientific measurement of candidates' experience during a job interview. EEG and skin conductance measurement allowed to identify the most engaging and stressful phases during a job interview. Moreover, this study allowed to identify differences in the interviewers' styles, showing that a quiet style produces less stress, allowing the candidate to conduce a performant interview, and allowing the interviewer to capture the candidates' potential. This study suggests implication for the assessment process and contribute to the neuromanagement understanding in the light of organizational psychology.

## Author Contributions

All authors contributed to the preparation and revision of the Editorial. All authors contributed to the article and approved the submitted version.

## Conflict of Interest

The authors declare that the research was conducted in the absence of any commercial or financial relationships that could be construed as a potential conflict of interest.

## Publisher's Note

All claims expressed in this article are solely those of the authors and do not necessarily represent those of their affiliated organizations, or those of the publisher, the editors and the reviewers. Any product that may be evaluated in this article, or claim that may be made by its manufacturer, is not guaranteed or endorsed by the publisher.
